# Clinical Neuropsychological Profile and Quality of Life in Women Who Have Suffered Gender-Based Violence

**DOI:** 10.1089/whr.2023.0019

**Published:** 2023-08-25

**Authors:** Alexandra Yakeline Meneses Meneses, Sol Fernandez-Gonzalo, Mercè Jodar Vicente

**Affiliations:** ^1^ Arts and Humanities Area, Psychology, Israel Technological University of Ecuador, Ecuador.; ^2^ Department of Psychology, Health District 17D10, Cayambe - Pedro Moncayo, Ecuador.; ^3^ Department of Clinical and Health Psychology, Universitat Autònoma de Barcelona, Barcelona, Spain.; ^4^ Instituto de Investigación e Innovación Parc Tauli-I3PT, Sabadell, Spain.; ^5^ CIBERSAM, Instituto de Salud Carlos III, Madrid, Spain.; ^6^ Neurology Service, Hospital Universitario Parc Tauli, Sabadell, Spain.

**Keywords:** gender-based violence, battered women, violence against women

## Abstract

**Background:**

This research characterizes the clinical and neuropsychological profiles and the quality of life in a group of Ecuadorian women who suffered physical violence, psychological violence, or sexual violence, exploring their relationships with sociodemographic factors.

**Methods:**

A battery of tests were used to explore the clinical and neuropsychological functions and quality of life in 120 participants who were selected from a population affected by violence.

**Results:**

Sixty percent of the participants showed clinical anxiety, 26.7% clinical depression, 40% post-traumatic stress disorder symptoms, 15% moderate personality disorder, and 51.7% a low quality-of-life index. Their Z-scores in the neuropsychological domains evaluated were verbal memory (Rey Auditory Verbal Learning Test = −1.35), working memory (Digits = −1.67), attention (D2 = −1.24), processing speed (Coding = −1.33; Trail Making Test A = 1.81), and executive function (Trail Making Test B = −1.15; Stroop = −0.20; verbal-semantic fluency test = 0.05; verbal fluency test = −1.23).

**Conclusions:**

The majority of women who suffered gender-based violence presented clinical levels of anxiety, depression, and post-traumatic symptoms. The cognitive functions with lower scores (*Z* < −1.5) were working memory and processing speed, mediated by education factor.

## Background

Violence against women, especially violence by their partners and sexual violence, constitutes a serious public health problem and a violation of the human rights of women. According to the World Health Organization,^1^ one in three women in the world has suffered physical and/or sexual violence by her partner or sexual violence by third parties at some point in their lives. There has been a recent increase in studies of how violence against women affects their health in different areas, including the mental,^2–4^ cognitive,^5,6^ physical,^7,8^ sexual,^9,10^ and social dimensions.^11–13^ Studies conducted in Ecuador indicate that 64.9% of women are victims of some type of violence,^14^ the most frequent being physical violence, psychological violence, and sexual violence.^15^

Authors, such as Kaur and Kumar^16^ and Bichard et al.,^17^ have pointed out that these violent actions can have permanent physical sequelae such as motor disorders, burns, and scars, reaching high levels of severity and leading to loss of consciousness or mild head trauma. Cognitive functioning, including functions such as attention and executive functions, may be secondarily affected.^18–20^ Sugg,^21^ reported other clinical symptoms associated with victims of violence: chronic pain, gastrointestinal disorders, obesity, chronic diseases, symptoms of insomnia, fatigue, and genitourinary infections. Sexually transmitted infections may appear, as well as serious consequences in the sexual and reproductive health of women as a result of the violent act.^22,23^

Another of the consequences observed is psychopathological alterations. The Pan American Health Organization (PAHO),^24^ mentions that the disorders with the highest incidence are affective mood disorders and those related to stress, as well as anxiety disorders,^25–27^ depression,^28–30^ personality disorders,^31–33^ and post-traumatic stress.^34,35^ Factors, such as age, marital status, level of education, and economic status, have been correlated with the severity of psychological symptoms and the increase in reported cognitive difficulties.^36–38^

Despite the aforementioned seriousness of the effects of gender-based violence on women, social support services, access to health services, accompaniment, and judicial protection have not shown efficiency and have not guaranteed the redignification of women and the rights of these victims, so the rapid and timely response of these services can have important implications on the recovery and well-being of women within their communities.^39,40^

Few studies have comprehensively evaluated the impact of violence, differentiating between the type of violence to which the victims have been exposed. That is why our research aims to examine the clinical and neuropsychological profile and the quality of life in Ecuadorian women who suffered different types of violence (physical, psychological, and sexual). We hypothesized that women who suffered gender violence would present high levels of anxiety, depression, post-traumatic stress symptoms, alterations in the personality profile, and low performance on standardized cognitive tests; and that sociodemographic characteristics, such as level of education, marital status, type of violence, and time of exposure to violence, would be correlated with the presence and severity of psychopathological and cognitive effects.

## Methods

### Participants

The study population was identified in the Communities of Tabacundo and Cayambe, by the psychology service of the Tabacundo Health Center—Ecuador. It should be noted that these are populations that show high rates of gender violence^14^; most of the families are dedicated to floriculture, and they come from indigenous communities. As part of the strategy for the prevention and eradication of violence against Ecuadorian women, the staff of the Psychology service of the Tabacundo Health Center—Ecuador, identified the users who declared having been exposed to violence in the automated system of consultations and outpatient care RDACAA—WEB, during the study period 2018 to 2019, reaching a total of 120 women exposed to gender-based violence, namely physical, psychological, and sexual.^41^

Within its sociodemographic characteristics we have the sample that corresponds to women from the communities of Cayambe and Pedro Moncayo in the Province of Pichincha—Ecuador, with an average age of (mean [M]: 34.3; standard deviation [SD]: 0.7), most of whom are under common law partnership, living with a male partner (59%).

Their average level of years of schooling is (M: 8.3; SD: 0.3), and with an average intelligence coefficient (M: 81.1; SD: 0.8); all of them have a history of exposure to gender-based violence, at least once, at any stage of their lives, whose typology reported as the most recurrent was physical (46%), psychological (43.33%), or sexual (10%). The average time of having been exposed to violence was (M: 6.6; SD: 0.5). The inclusion criteria were as follows: (1) exposure to gender violence (physical, psychological, and/or sexual); (2) age between 18 and 50 years; and (3) agreement to voluntarily participate and having signed the informed consent form. The exclusion criteria were as follows: (1) intelligence quotient (IQ) less than 70; (2) history of acquired brain damage; (3) history of severe neurological or psychiatric illness; (4) history of toxic consumption habits; and (5) presence of sensory alterations that impede the performance of the tests.

### Procedure

The research was approved by the health committee of District 17D10, Cayambe—Pedro Moncayo, and by the Ethics and Research Committee of the UTE University. The Mental Health staff of the Tabacundo Health Center and external collaborating researchers of the Parc Taulí Hospital in Barcelona participated. The women were selected to be part of the study under inclusion and exclusion criteria. All participants were previously informed of the characteristics and procedures of the study and of the voluntary nature of their participation and signed the informed consent form. Women who agreed to participate in the study were first administered the National Adult Reading Test NART test^42^ to rule out intellectual disability. All women who met the inclusion criteria met with the study psychologist for the assessment of their psychopathological and cognitive status. The evaluation lasted at most 2 hours and consisted of the application of a wide battery of tests and questionnaires to evaluate the psychoaffective, cognitive, and quality-of-life dimensions.

#### Instruments

##### Survey on gender violence

The survey on gender violence was based on the Technical Standard for Comprehensive Attention to Gender Violence. It allows collecting data associated with sociodemographic variables, age, gender, marital status, and economic status, as well as the type of reported violence and the time of exposure to violence.

##### National adult reading test NART

This is a test for reading unstressed words, which correlates with the QI index of the WAIS intelligence test^42–44^ and allows estimating the level of QI. The premorbid IQ is scored according to direct form of 0/30, a score of 10 on NART equals 31 on WAIS-IV, and a score of 30 equals 136 on WAIS-IV. Cronbach's alpha coefficient of validity is 0.84.^44^

### Psychopathological/clinical measures

#### Hospital Anxiety and Depression Scale

It allows to assess the levels of anxiety and depression that the subject presents.^45^ It consists of seven items related to anxiety symptoms and seven items related to depressive symptoms, and each item is valued on a scale of 0 to 3 points. The cut point is 7. Subscale scores for anxiety: 0–7: No anxiety; 8–10: Minimal anxiety; and 11–21: Clinical anxiety. Subscale scores for depression: 0–7: No depression; 8–10: Minimum depression; 11–21: Clinical depression. Cronbach's alpha coefficient of validity is 0.76.

#### Post-traumatic Stress Disorder Checklist for The Diagnostic and Statistical Manual of Mental Disorders, Fifth Edition

It is an instrument updated to the The Diagnostic and Statistical Manual of Mental Disorders, Fifth Edition (DSM-5), to evaluate post-traumatic symptoms.^46,47^ Respondents indicate how much each post traumatic stress disorder (PTSD) symptom has disturbed them in the past week (as opposed to the past month), using a 5-point scale ranging from 0 = not at all, 1 = a little, 2 = moderately, 3 = quite a bit, and 4 = extremely. The cut point is 31. Note 1: The patient must previously meet Criterion A. Note 2: A total score of 31 to 33 points is optimal to determine probable PTSD. Note 3: To determine whether or not it meets the Criterion for a Provisional Diagnosis PTSD, we will only consider symptoms with scores of 2, 3, or 4. Cronbach's alpha coefficient of validity is 90 to 0.97.

##### Millon Clinical Multiaxial Inventory—IV

The scales corresponding to Axis 2 (Clinical Patterns of Personality and severe personality pathology) were considered.^48^ Base Rate Profile is as follows: Validity scales: 0–35: low; 36–74: medium; and 75–100: high. Clinical patterns of personality: 0–60: no risk; 61–74: style; 75–84: type; and 85–115: personality disorder. Clinical syndromes: 0–74: absent; 75–84: present; and 85–115: prominent. Cronbach's alpha coefficient of validity is 0.72 to 0.82.

#### GENCAT Quality of Life Scale

It evaluates the quality-of-life index of the users, in the dimensions: emotional well-being, interpersonal relationships, material well-being, personal development, physical well-being, self-determination, social inclusion, and rights.^49^ The items of each dimension are stated using a frequency scale of four options. Standard scores (*M* = 10; SD = 3) of each Quality dimension of Life; percentiles and Quality of Life Index; Quality of life index of 52 < 1 percentile; and Quality of life index of 138 > 99 percentile. Cronbach's alpha coefficient of validity is 0.91.

### Neuropsychological measures

#### The Rey Auditory Verbal Learning Test

It measures verbal learning ability and short- and long-term retention. A target list of 15 words is read to the patient, who must try to remember it during 5 consecutive trials. Subsequently, a distracting list is presented, and the short-term memory of the target list is counted. After 30 minutes, long-term memory of the words on the list is collected. The adaptation of Ferreira for the Latin American population was applied.^50^ The maximum value is 15 and the minimum value is 0. Means of references by age and schooling were controlled, based on the reference population. Verbal learning and memory are assessed by the learning curve, the total acquisition or total learning (SI–V) and trials V (final acquisition level), VII (delayed recall), and VIII (recognition). Cronbach's alpha coefficient of validity is 0.80.

#### Digits-WAIS-IV

It is used to measure immediate memory and working memory capacity.^51^ It consists of repeating a series of numbers from less to greater complexity, it starts in a sequential order, then in reverse order and finally in increasing order. WAIS-IV scales were used, according to age. A score below 7 represents limited cognitive function. Cronbach's alpha coefficient of validity is 0.94.

#### Coding—subtest WAIS-IV

The task consists of completing with the appropriate symbols, some squares that have a digit in their upper part.^51^ It evaluates, the speed and visual motor skills. WAIS-IV scales were used, according to age. A score below 7 represents limited cognitive function. Cronbach's alpha coefficient of validity is 0.94.

#### Test D2

It measures selective and sustained attention.^52^ The test contains 14 lines with 47 characters, totalizing 658 items. These stimuli contain the letters “d” or “p” that can be accompanied by one or two small lines located, individually or in pairs, at the top or bottom of each letter. The subject's task consists of carefully reviewing the content of each line from left to right, then marking every letter “d” that has the assigned consignment or key, discriminating the relevant elements from the irrelevant ones. The direct scores obtained in Overall Test Efficiency (M: 430.71; SD: 99.75), concentration index (M: 172.64; SD: 48.30), and variation index (M: 14.57; SD: 6.13) were considered, according to reference population. Cronbach's alpha coefficient of validity is 0.80–0.97.

#### Trail Making Test, part A and part B

This test allows assessing visual motor speed and cognitive flexibility.^53^ Part A consists of executing a task, joining, without releasing the line, numbers in chronological order from 1 to 25. Part B consists of executing another more complex task, in which numbers and letters must be joined in order of alternate increasing (1 a, 2 b … 13. in sequential order) under time pressure, and the number of mistakes made is counted. A standardized version for the Latin population was used.^53^ The direct scores obtained in seconds were considered, according to the means obtained in the reference population, under control of age and schooling. Cronbach's alpha coefficient of validity is 0.70 to 0.90.

#### Stroop test

It is an instrument that assesses complex attention, through the ability to inhibit verbal interference, or faster automatic response, so it is a good measure of selective attention.^54^ The level of interference was obtained, based on the scores obtained that mark a typical score of 20 to 80. The results were interpreted, according to the means obtained in the reference population. Cronbach's alpha coefficient of validity is 0.89.

#### Phonological verbal fluency test

It consists of a controlled and programmed verbal production test, in which the subject must produce words that begin with a letter pre-established by the examiner for 1 minute (example: P—M—R).^55^ The scores were obtained directly, and were analyzed based on the means obtained in the reference population. Cronbach's alpha coefficient of validity is 0.82.

#### Semantic verbal fluency test

It assesses the ability to retrieve stored semantic information.^55^ It is related to the speed to organize the thought and the strategies used for the fast search of words, in a time of 60 seconds. The scores were obtained directly, and were analyzed based on the means obtained in the reference population. Cronbach's alpha coefficient of validity is 0.82.

### Statistical analysis

SPSS v24.0.0 and R v3.6.2 were used for data processing. A descriptive analysis of the data was performed to present the sociodemographic profile, reporting the mean and SD. The direct scores of the cognitive tests were transformed to z scores to place the women studied with respect to the standard averages that are offered by the scales of each test and that consider sex, age, and schooling. *Z* < −1.5 was set as the cutoff point to determine the cognitive impairment of clinical relevance in the cognitive tests.^56^ Analysis of variance and Kruskal−Wallis tests were used, according to the fulfillment of the statistical test requirements, to analyze the differences between types of violence (physical, psychological, and sexual violence) according to disaggregation groups (sociodemographic factors); to demonstrate or not the existence of a significant difference, multiple contrast tests (*post hoc*) were constructed. In addition, the type of tests used in the data analysis plan for multiple comparisons are added as shown in the Supplementary Material (Appendix A2).

## Results

No differences in sociodemographic variables were observed between the groups who suffered physical, psychological, and sexual violence, except that the group with sexual violence showed a higher IQ than the group with sexual, physical, or psychological violence (*F* = 4, 40; *p* = 0.014) (Table 1).

**Table 1.** Sociodemographic Characteristics of the Participants

Variables	Total	Physical violence	Psychological violence	Sexual violence	***p***-valor
*N*	120	56	52	12	
Age, M: SD	34.3: 0.7	35.4: 1.1	33.6: 1.1	32.3: 2.9	0.341
Years of schooling, M: SD	8.3: 0.3	7.8: 0.4	8.7: 0.5	8.8: 0.9	0.301
Violence exposure time, M: SD	6.6: 0.5	7.2: 0.8	6.2: 0.7	5.7: 1.3	0.583
IQ, M: SD	81.1: 0.8	81: 0.9	80: 1.1	88: 4.0	0.014^*^
Marital status, *n* (%)					
Single	16 (100)	6 (37.5)	7 (43.8)	3 (18.8)	0.504
Married	38 (100)	19 (50.0)	15 (39.5)	4 (10.5)	0.504
Divorced	7 (100)	5 (71.4)	1 (14.3)	1 (14.3)	0.504
Free union	59 (100)	26 (44.1)	29 (49.2)	4 (6.8)	0.504

^*^ *p* = < 0.05 Differences in the averages obtained in the estimated IQ in the groups by types of violence.

IQ, intelligence quotient; M, mean; SD, standard deviation.

**FIG. 1.**
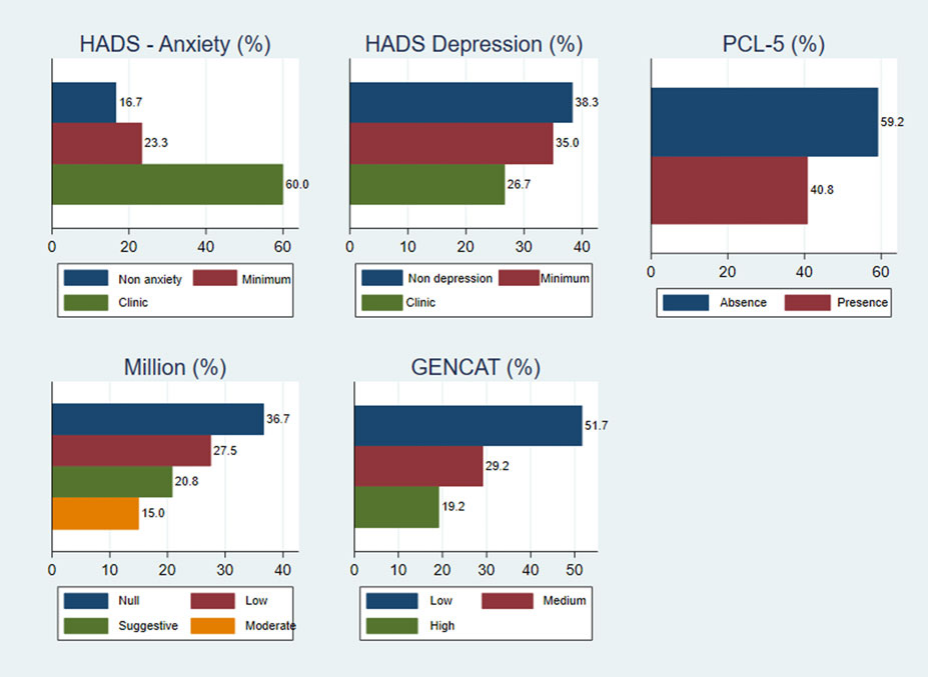
Percentages obtained in the tests that indicate the presence or absence of clinical/psychopathological alterations in the global simple. Millon: Millon Clinical Multiaxial Inventory-IV; GENCAT: GENCAT Quality of Life Scale; DSM-5, The Diagnostic and Statistical Manual of Mental Disorders, Fifth Edition; HADS-Anxiety, Hospital Anxiety and Depression Scale (Anxiety); HADS Depression, Hospital Anxiety and Depression Scale (Depression); PCL-5, Post-traumatic Stress Disorder Checklist for DSM-5.

The general results of the clinical tests administered indicate that 60.0% of women who suffered violence presented clinical anxiety (Hospital Anxiety and Depression Scale [HADS]-A), 36.7% reported clinical depression (HADS-D), and 40.8% of the women evaluated presented post-traumatic stress symptoms (Post-Traumatic Stress Disorder Checklist for DSM-5 (PCL-5). Regarding the personality profile (Million-III), 15.0% had a moderate risk of pathology or personality disorder, and 51.7% had a low quality-of-life index (GENCAT) (Fig. 1).

**FIG. 2.**
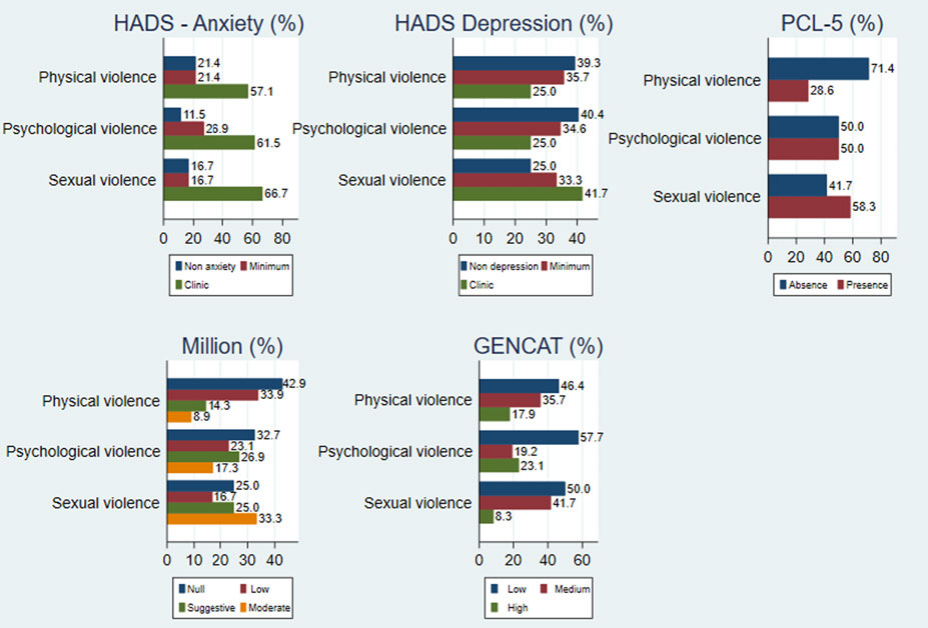
Results obtained from the tests applied in the clinical/psychopathological variables by the type of violence.

When comparing the values obtained on the different tests applied, divided by type of violence suffered, those women who suffered sexual violence more often had clinical anxiety (66.7%), clinical depression (41.7%), post-traumatic stress (58%), moderate-risk personality disorder (42.9%), and low quality of life (50%) than those women who reported physical and psychological violence (Fig. 2). No correlations were found between the clinical malady and the type of violence, except that post-traumatic stress was more common in the group of women who suffered sexual violence (*p* = 0.003) (Table 2). This nonsignificant difference was confirmed with the *post hoc* tests carried out, presenting some of them in confidence interval graphs for means (Appendix 3).

**Table 2.** Differences Among Groups of Cognitive, Clinical, and Sociodemographic Variables

Variables	Test	Age		ANOVA	Years of schooling		ANOVA	Living without a partner	Living with a partner	Kruskal–Wallis	Physical violence (***n*** = 56)	Psychological violence (***n*** = 52)	Sexual violence (***n*** = 12)	Kruskal–Wallis
		**18 to 30 years old *n* = 37**	**31 to 50 years old *n* = 83**		**<8 years old (*n* = 70)**	**>8 years old (*n* = 50)**		**(*n* = 23)**	**with a partner (*n* = 97)**					
		**M;DS**	**M;DS**		**M;DS**	**M;DS**		**M; DS**	**M; DS**		**M;DS**	**M; DS**	**M; DS**	
Verbal memory	RAVLT	−0.97; 0.68	−1.26; 0.58	0.606	−1.25; 0.66	−1.06; 0.65	0.253	−0.88; 0.58	−1.31; 0.55	0.354	−1.24; 0.56	−1.11; 0.55	−1.09; 0.55	0.726
Working memory	Digits	−1.28; 0.60	−1.43; 0.66	0.384	−1.47; 0.63	−1.55; 0.66	0.078	−1.58; 0.60	−1.47; 0.65	0.599	−1.26; 0.63	−0.84; 0.64	−0.81; 0.63	0.392
Attention	D2	−1.07; 0.64	−1.51; 0.65	0.200	−1.54; 0.66	−1.14; 0.65	**0.002** ^*^	−1.00; 0.66	−1.67; 0.65	0.137	−1.17; 0.65	−1.47; 0.60	−1.96; 0.58	0.082
Processing speed	Coding	−1.43; 1.68	−1.34; 1.55	0.649	−1.74; 0.87	−0.84; 0.72	0.190	−1.54; 0.78	−1.28; 0.75	0.836	−1.42; 0.75	−1.31; 0.70	−1.42; 0.65	0.836
Executive functions	TMT-B	−1.11; 0.63	−1.20; 0.65	0.492	−1.80; 0.79	−1.38; 0.75	0.749	−2.17; 0.60	−1.59; 0.66	0.177	−1.10; 0.65	−1.43; 0.65	−2.40; 0.68	0.174
	Stroop	−0.85; 0.60	−0.60; 0.54	0.812	−1.58; 0.64	−1.45; 0.65	0.741	−1.17; 0.64	−1.51; 0.65	0.281	−0.21; 0.64	−0.28; 0.64	0.23; 0.65	0.279
	SVFT	0.03; 0.65	0.02; 0.65	0.292	−0.89; 0.58	−0.79; 0.55	0.478	−0.66; 0.52	−0.5; 0.55	0.545	0.13; 0.57	0.16; 0.55	0.19; 0.54	0.737
	PVFT	−1.04; 0.63	−1.23; 0.91	0.366	−1.22; 0.57	−1.10; 0.60	0.751	−1.44; 0.64	−0.74; 0.65	0.231	−1.28; 0.63	−1.03; 0.65	−0.98; 0.68	0.501
Anxiety	HADS-A	11.70; 4.55	11.3; 4.24	0.309	11.32; 3.82	11.68;4.50	0.346	10.80; 4.52	12.39; 4.40	**0.012** ^**^	10.86; 4.00	11.9; 3.50	12.5; 3.50	0.335
Depression	HADS-D	8.29; 3.63	8.5; 4.03	0.903	8.68; 4.09	8.22; 3.60	0.326	9.06; 3.40	9.18; 3.30	0.125	8.2; 3.20	8.48; 3.30	9.92; 3.60	0.474
Post-traumatic stress	PCL-5	50.12; 5.25	50.4; 5.25	0.162	49.63; 7.89	51.4; 7.77	0.423	31.06; 7.75	30.63; 7.20	0.136	30.6; 7.30	34.0; 6.60	50.8; 6.70	**0.003** ^***^
Personality	Millon	46.16; 4.53	45.3; 9.41	0.317	44.78; 3.50	46.7; 4.30	0.428	62.25; 4.30	49.34; 4.10	0.070	45.03; 4.30	48.5; 3.40	54.25; 3.5	0.068
Quality of life	GENCAT	94.24; 16.07	94.9;15.07	0.156	93.27; 15.78	96.8; 15.76	0.423	96.33; 14.50	92.97; 13.70	0.289	95.13; 14.30	94.5;13, 7	93.9; 14.1	0.916

Digits: WAIS-IV Digits subtest; D2: Test D2; Coding: WAIS-IV Coding subtest; Stroop: Stroop Test; Millon: Millon Clinical Multiaxial Inventory-IV.

^*^ *p*-Value calculated using ANOVA (analysis of variance) to compare differences between groups by years of schooling, in the averages estimated in clinical and neuropsychological tests.

^**^ *p*-Value calculated using Kruskal Wallis to compare differences between groups by marital status, in the averages estimated in clinical and neuropsychological tests.

^***^ *p*-Value calculated using Kruskal Wallis to compare differences between groups by type of violence, in the averages estimated in clinical and neuropsychological tests.

ANOVA, analysis of variance; DSM-5, The Diagnostic and Statistical Manual of Mental Disorders, Fifth Edition; GENCAT: GENCAT Quality of Life Scale; HADS-Anxiety, Hospital Anxiety and Depression Scale (Anxiety); HADS Depression, Hospital Anxiety and Depression Scale (Depression); PCL-5, Post-Traumatic Stress Disorder Checklist for DSM-5; PVFT, phonetic verbal fluency test; RAVLT, The Rey Auditory Verbal Learning Test; SVFT, semantic verbal fluency test; TMT-B, Trail Making Test Part B.

**FIG. 3.**
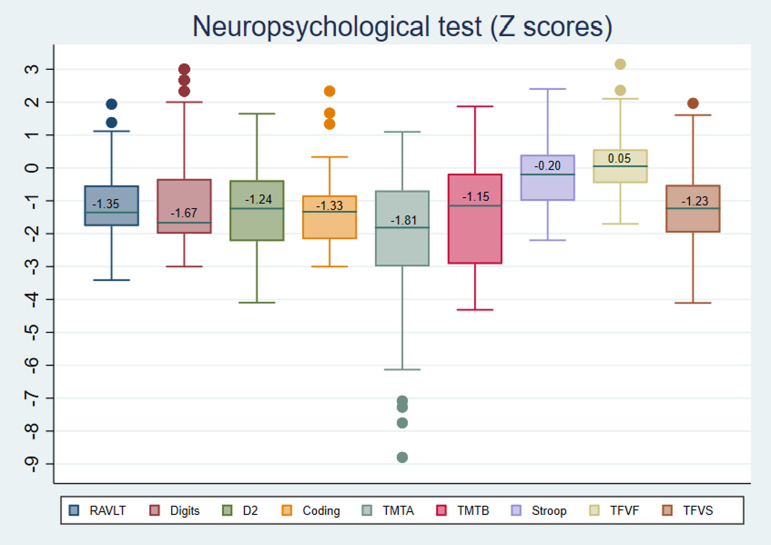
Z score distribution of the tests that assess neuropsychological functions. Digits: WAIS-IV Digits subtest; D2: Test D2; Coding: WAIS-IV Coding subtest; Stroop: Stroop Test; RAVLT, The Rey Auditory Verbal Learning Test; PVFT, phonetic verbal fluency test (TFVF: Spanish version); TMT-B, Trail Making Test Part B; SVFT, semantic verbal fluency test; (TFVS: Spanish versión).

Regarding the neuropsychological variables, a clinically significant decrease was only observed in the working memory variable (Digits = −1.67) and in the processing speed variable (Trail Making Test part A [TMT-A] = −1.81). It is important to mention that, although no clinically significant results were observed in the rest of the averages, there was a tendency to present values lower than the average for their age and cultural level of verbal memory (Rey Auditory Verbal Learning Test = −1.35), attention (D2 = −1.24), visual motor speed (Coding = −1.33), and executive function tasks (Trail Making Test part B [TMT-B] = −1.15, Stroop = −0.20, phonetic verbal fluency with phonetic instruction = 0.05, and verbal fluency with semantic command = −1.23) (Fig. 3).

**FIG. 4.**
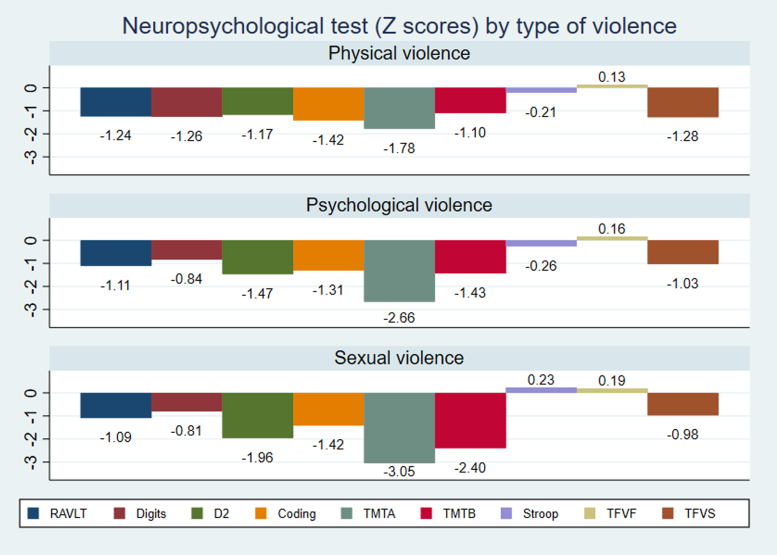
Differences among the groups of physical, psychological, and sexual violence studied, regarding the reported neuropsychological performance.

Comparing the means of the scores on the neuropsychological tests between the groups of physical, psychological, and sexual violence, the group of women who suffered sexual violence had clinically significant scores (*Z* < −1.5) on the screening tests (TMT-A and TMT-B) and in the attention test (D2) compared with the averages among the physical and psychological violence groups, which were slightly but not significantly below the average for their age and schooling level (Fig. 4).

The differences in the scores between the groups that differed by cognitive and clinical variables were mediated by the sociodemographic factor. Women with <8 years of schooling showed lower scores on the attentional tests than women with >8 years of schooling (D2, *p* = 0.02). The Kruskal–Wallis test showed that women who suffered sexual violence more often had post-traumatic symptoms than women who suffered physical and psychological violence (PCL-5, *p* = 0.003). Women who lived with their romantic partner showed greater anxiety than women who did not live with a partner (HADS-A, *p* = 0.012) (Table 2). Weak correlations were found between the scores on the neuropsychological tests and the clinical variables (Table 3).

**Table 3.** Relationship Between Cognitive and Clinical Variables

Variable	Test	HADS-A		HADS-D		PCL-5		MILLON		GENCAT	
		Pearson's R	***p***	Pearson's R	***p***	Pearson's R	***p***	Pearson's R	***p***	Pearson's R	***p***
Verbal memory	RAVLT	0.045	0.625	0.18	0.849	−0.104	0.258	0.072	0.436	−0.104	0.258
Working memory	Digits	0.162	0.078	−0.126	0.169	0.108	0.241	−0.048	0.599	0.108	0.241
Attention	D2	−0.024	0.794	−0.156	0.088	−0.063	0.497	−0.143	0.118	−0.063	0.497
Processing speed	Coding	0.010	0.914	−0.162	0.076	0.038	0.681	−0.001	0.988	0.038	0.681
	TMT-A	−0.067	0.465	0.066	0.477	−0.133	0.148	0.145	0.115	−0.133	0.148
Executive functions	TMT-B	−0.129	0.162	−0.037	0.690	0.002	0.980	−0.057	0.535	0.002	0.980
	Stroop	−0.004	0.963	0.074	0.423	−0.036	0.699	−0.008	0.935	−0.036	0.699
	PVFT	0.101	0.273	−0.028	0.757	0.033	0.721	0.056	0.540	0.033	0.721
	SVFT	0.052	0.575	−0.095	0.303	0.074	0.419	0.047	0.611	0.074	0.419

## Discussion

To the best of our knowledge, this is the first study that evaluates the impact of violence against women, in the cognitive, psychoaffective, and social dimensions, and explores its association with sociodemographic factors. As main results of the study, it was found that women exposed to gender violence, domiciled in Cayambe and Pedro Moncayo, Ecuador, present clinical levels of anxiety, depression and post-traumatic stress, and a decrease in quality of life.

The effects of violence showed an association with some sociodemographic factors, thus, women who live with their sentimental partner presented higher levels of anxiety, compared with women who live without a sentimental partner. In addition, women exposed to sexual violence presented a significantly higher level of PTSD symptoms and obtained a low cognitive profile, with difficulties in attention tasks and executive functions. In addition, sociodemographic characteristics, such as level of education, marital status, type of violence, and length of exposure to violence, are related to the presence and severity of psychopathological conditions, especially levels of anxiety and post-traumatic stress.

In this sense, the results obtained in this research partially support the initial hypothesis, which had assumed that the psychological profile of women exposed to violence would be affected. Such affectation is repeated in stories of women violated in other cultural and social settings, where, according to the studies, sequels for mental health were reported, such as anxiety disorders,^25–27^ depressive disorders,^28–30^ affectation in the personality profile,^31–33^ and post-traumatic stress.^3,4,34,35^

Our study suggests that violence against women not only affects mental health, but also affects performance in activities of daily living, which require attention and concentration, as well as negatively impacts social functioning, decreasing the quality of life of women.^18–20^ Therefore, it is based on what has already been mentioned by the WHO^1,24^ that violence against women is not only a global social problem, but also a public health one.

It is also important to highlight in our findings that sexual violence has greater repercussions compared with physical and psychological violence, especially in the presence of post-traumatic symptoms. Previous studies had already warned about the impact of sexual violence on the physical and mental health of the victims.^34,35^

Also, it was observed that the group of women exposed to sexual violence presented clinically significant scores (*z* < −1.5) in the tasks of sustained attention, processing speed, and executive functions. This finding is consistent with other populations of sexually abused women, who showed greater impairment in verbal memory, executive functions, and attention, compared with women who had not been subjected to sexual violence.^10,56^

As it has already been shown that health is a determinant of quality of life, in this sense, this study was able to verify that more than 50% of women exposed to violence presented a low level of quality of life. These findings are consistent with data reported in other contexts, both in developed and developing countries. This risk pattern seems to be characteristic among our women, regardless of their nationality and/or cultural group they belong to.^11–13,57–60^

These findings are important for organizations that design support programs for victims of violence, for a timely global intervention and rehabilitation that can generate an impact on the maximum possible number of women, in accordance with the objectives of sustainable development. The results of this study support the need for a comprehensive assessment, not only physical but psychological and social in victims of violence, encouraging a rethinking of reception and intervention protocols, with special focus on care for women victims of sexual violence.

## Limitations

This study is not without limitations. First, it was not possible to include a control group of women not exposed to violence to study the differences from women who suffered violence. Second, the low level of education of the women in this study and the fact that they all belonged to the same floriculture community does not allow the results to be generalized to other populations of women with other socioeconomic levels.

Women with a history of cranioencephalic trauma and toxic consumption habits were excluded from this study, due to the fact that a neuropsychological battery was used, which is not adapted for these populations. Therefore, the rates of psychopathology reported in this article may have been underestimated.

Finally, exposure to trauma can be ongoing and multifaceted, so the categorization of trauma in the study problem presented examines only one episode of care (exposure to events related to gender-based physical, psychological, and sexual violence). Therefore, there are limitations in capturing the complexity of the possible psychological consequences and effects on the quality of the victims, since the reported symptoms could, in addition to the traumatic event of exposure to gender-based violence, also derive from many different traumas and/or early traumas that persisted over time.

## Conclusions

In general, the majority of women exposed to gender-based violence who participated in this study, showed considerable levels of anxiety, depression, and post-traumatic symptoms, as well as poor performance in the functions of verbal memory, attention, processing speed and executive function, in addition to a low level of quality of life.

Regarding the differences between the groups of cognitive and clinical variables, mediated by the sociodemographic factor, it was found that the group of women exposed to sexual violence presented a higher level of post-traumatic stress, and clinically significant negative scores on attention tests sustained, processing speed and executive capacity, compared with the groups of women exposed to physical and psychological violence. Also, the women who live with their sentimental partner showed a higher level of anxiety, compared with those who live without a partner.

The integration of the results presented a closer picture of the physical, psychological, and social damage in the victims of violence, and suggests a reconsideration of the reception and intervention protocols, integrating the sociodemographic factor that accompanies each affected population, and prioritizing women victims of sexual violence.

## Supplementary Material

Supplemental data

## Data Availability

The data that support the findings of this study are available from the corresponding author upon reasonable request.

## Abbreviations Used

ANOVA: analysis of variance
DSM-5: The Diagnostic and Statistical Manual of Mental Disorders, Fifth Edition
GENCAT: GENCAT Quality of Life Scale
HADS-Anxiety: Hospital Anxiety and Depression Scale (Anxiety)
HADS Depression: Hospital Anxiety and Depression Scale (Depression)
IQ: intelligence quotient
M: mean
NART: National Adult Reading Test
PCL-5: Post-Traumatic Stress Disorder Checklist for DSM-5
PVFT: phonetic verbal fluency test (TFVF: Spanish version)
RAVLT: Rey Auditory Verbal Learning Test
SD: standard deviation
SVFT: semantic verbal fluency test (TFVS: Spanish versión)
TMT: Trail Making Test
