# Supplementary material for: Clinical Neuropsychological Profile and Quality of Life in Women Who Have Suffered Gender-Based Violence
Source: Womens Health Rep (New Rochelle). 2023 Aug 25;4(1):448–60. doi: 10.1089/whr.2023.0019 (PMC10460961; doi:10.1089/whr.2023.0019)
Supplement: Supplemental data [file Suppl_Data.docx]

**Supplementary material**

**Appendix 1**

**Table 1**

*Description of the tests*

| **Test** | **Diagnostic Scale** | **Cronbach's Alpha** |
| --- | --- | --- |
| Hospital Anxiety and Depression Scale HADS (Zigmond y Snaith, 1983). | Subscale scores for anxiety:  0-7: No anxiety  8-10: Minimal anxiety  11-21: Clinical anxiety  subscale scores for depression:  0-7: No depression  8-10: Minimum depression  11-21: Clinical depression | .76 - .88 |
| Posttraumatic Stress Disorder Checklist for DSM-5 (Blevins et al., 2015; Di Tella et al., 2022). | Note 1. The patient must previously meet Criterion A.  Note 2. A total score of 31 to 33 points is optimal to determine probable PTSD.  Note 3. To determine whether or not it meets the Criterion for a Provisional Diagnosis  PTSD, we will only consider symptoms with scores of 2, 3, or 4. | .90 to .97 |
| MCMI-IV (2018) | **Base Rate Profile**  *Validity scales*  0 – 35: low  36 – 74: medium  75 – 100: high  *Clinical patterns of personality*  0-60: no risk  61-74: style  75-84: type  85-115: personality disorder  *Clinical syndromes*  *0-74: absent*  *75 – 84: present*  *85 – 115: prominent* | .72 to .82 |
| GENCAT (Verdugo et al., 2009). | Standard scores (M= 10; SD= 3) of each Quality dimension  of Life, percentiles and Quality of Life Index.  Quality of life index of 52 <1 percentile  Quality of life index of 138 >99 percentile | .91 |
| National Adult Reading test NART/ TAP (Pluck et al., 2017); original version;  (Nelson & Willison, 1991); validity coefficient (Bright et al., 2016). | The premorbid intelligence quotient is scored according to direct form of 0/30  A score of 10 on NART equals 31 on WAIS-IV, and a score of 30 equals 136 on WAIS-IV | .84 |
| The Rey Auditory Verbal Learning Test RAV LT (Ferreira et al., 2013). | The maximum value is 15 and the minimum value is 0.  Means of references by age and schooling were controlled, based on the reference population.  Verbal learning and memory are assessed by the learning curve, the total acquisition or total learning (SI–V) and trials V (final acquisition level), VII (delayed recall), and VIII (recognition). | .80 |
| Digits direct and indirect subtests  WAIS-IV (Wechsler, 2012). | WAIS-IV scales were used, according to age  A score  A scale score below 7 represents limited cognitive function. | .94 |
| Cooding subtest  WAIS-IV (Wechsler, 2012). | WAIS-IV scales were used, according to age  A score  A scale score below 7 represents limited cognitive function. | .94 |
| D2 (Brickenkamp, 2012). | The direct scores obtained in TOT (M: 430.71; SD: 99.75), CON (M: 172.64; SD: 48.30), VAR (M: 14.57; SD: 6.13) were considered. According to reference population. | .80 - .97 |
| TMT (Margulis et al., 2018; Reitan, 1958;  Arango et al., 2015). | The direct scores obtained in seconds were considered, according to the means obtained in the reference population, under control of age and schooling. | .70 to .90 |
| Stroop Test (Golden, 2007). | The level of interference was obtained, based on the scores obtained that mark a typical score of 20 to 80. The results were interpreted, according to the means obtained in the reference population. | 0.89 |
| Verbal fluency test: phonetics and semantics (Artiola et al., 1999; Peña et al., 2009; Marquine et al., 2020). | The scores were obtained directly, and were analyzed based on the means obtained in the reference population. | 0.82 |

**Note.** TAP: The Word Accentuation Test (Spanish version); TOT: Overall Test Efficiency; CON: concentration index; VAR: variation index.

**Appendix 2**

**Table 2**

*Sociodemographic characteristics of the participants*

| Variables | Total | Physical violence | Psychological violence | Sexual violence | p-valor |
| --- | --- | --- | --- | --- | --- |
|  |  |  |  |  |  |
| N | 120 | 56 | 52 | 12 |  |
| Age (M: SD) | 34,3; 0,7 | 35,4; 1.1 | 33,6; 1,1 | 32,3; 2,9 | 0,341 |
| Years of schooling  (M: SD) | 8,3; 0,3 | 7,8; 0,4 | 8,7; 0,5 | 8,8; 0,9 | 0,301 |
| Violence exposure time (M: SD) | 6,6; 0,5 | 7,2; 0.8 | 6,2; 0,7 | 5,7; 1,3 | 0,583 |
| Intelligence quotient | 81,1; 0,8 | 81; 0,9 | 80; 1,1 | 88; 4,0 | 0,014 |
| (M: SD) |  |  |  |  |  |
| Marital status (N, %) | | | | | |
| Single | 16; 100 | 6; 37,5 | 7. 43,8 | 3, 18,8 | 0,504 |
| Married | 38; 100 | 19; 50,0 | 15; 39,5 | 4; 10,5 | 0,504 |
| Divorced | 7; 100 | 5; 71,4 | 1; 14,3 | 1; 14,3 | 0,504 |
| Free union | 59; 100 | 26; 44,1 | 29; 49,2 | 4; 6,8 | 0,504 |

**Figure 1**

*Age (M: SD)*


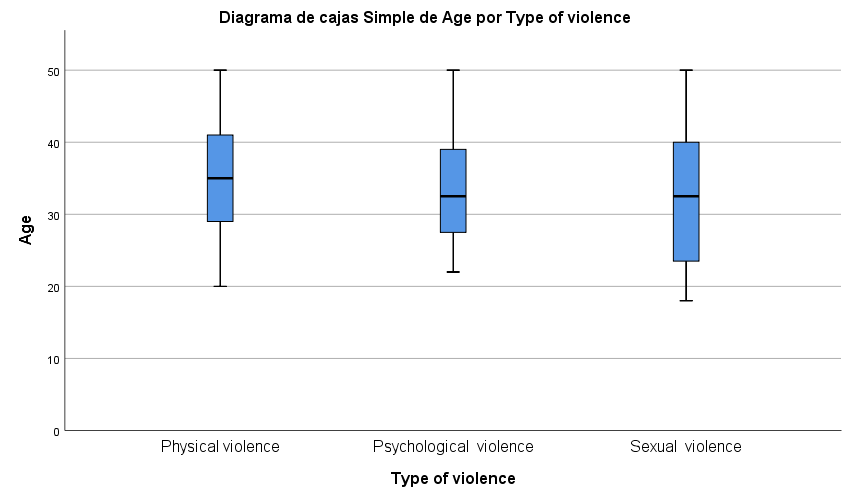


The p-value obtained is greater than 0.05 (p value > 0.05), so there are no significant differences between the types of violence, in relation to age. The post hoc test indicated is the one obtained by performing the ANOVA (Table 3).

**Table 3**

*Post hoc test Age – Type of violence*

| **Multiple comparisons** | | | | | | |
| --- | --- | --- | --- | --- | --- | --- |
| Dependent variable: | Age | | | | | |
| HSD Tukey | | | | | | |
| (I) Type of violence | | Difference of means (I-J) | Error deviation | Sig. | Confidence interval 95% | |
|  |  |  |  |  | Lower limit | Upper limit |
| Physical violence | Psychological violence | 1,794 | 1,573 | 0,491 | -1,94 | 5,53 |
|  | Sexual violence | 3,179 | 2,599 | 0,442 | -2,99 | 9,35 |
| Psychological violence | Physical violence | -1,794 | 1,573 | 0,491 | -5,53 | 1,94 |
|  | Sexual violence | 1,385 | 2,617 | 0,857 | -4,83 | 7,60 |
| Sexual violence | Physical violence | -3,179 | 2,599 | 0,442 | -9,35 | 2,99 |
|  | Psychological violence | -1,385 | 2,617 | 0,857 | -7,60 | 4,83 |

**Figure 2**

*Years of schooling*


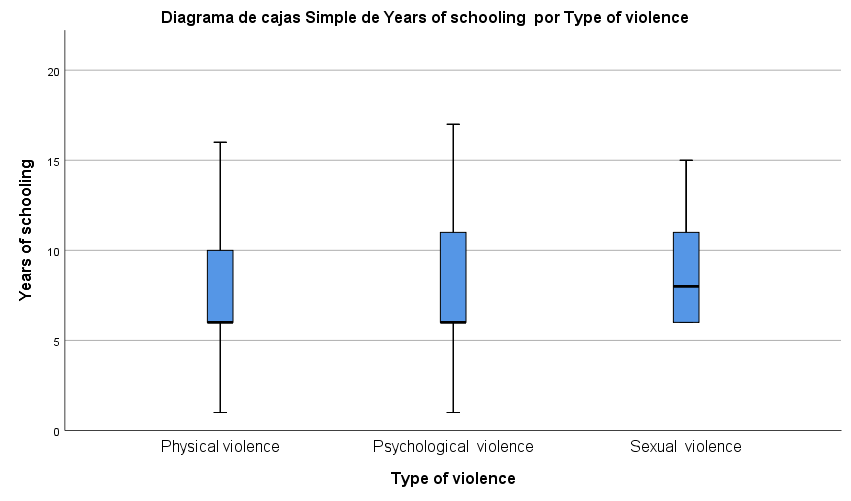


The p-value obtained is greater than 0.05 (p value>0.05), so there are no significant differences between the types of violence, in relation to the years of schooling. Next, the following post hoc test (Table 4).

**Table 4**

*Post hoc Test Years of schooling – Type of violence*

| **Multiple comparisons** | | | | | | |
| --- | --- | --- | --- | --- | --- | --- |
| Dependent variable: | Years of schooling | | | | | |
| HSD Tukey | | | | | | |
| (I) Type of violence | | Difference of means (I-J) | Error deviation | Sig. | Confidence interval 95% | |
|  |  |  |  |  | Lower limit | Upper limit |
| Physical violence | Psychological violence | -0,942 | 0,657 | 0,327 | -2,50 | 0,62 |
|  | Sexual violence | -1,083 | 1,086 | 0,580 | -3,66 | 1,49 |
| Psychological violence | Physical violence | 0,942 | 0,657 | 0,327 | -0,62 | 2,50 |
|  | Sexual violence | -0,141 | 1,093 | 0,991 | -2,74 | 2,45 |
| Sexual violence | Physical violence | 1,083 | 1,086 | 0,580 | -1,49 | 3,66 |
|  | Psychological violence | 0,141 | 1,093 | 0,991 | -2,45 | 2,74 |

**Figure 3**

*Violence exposure time (M: SD)*


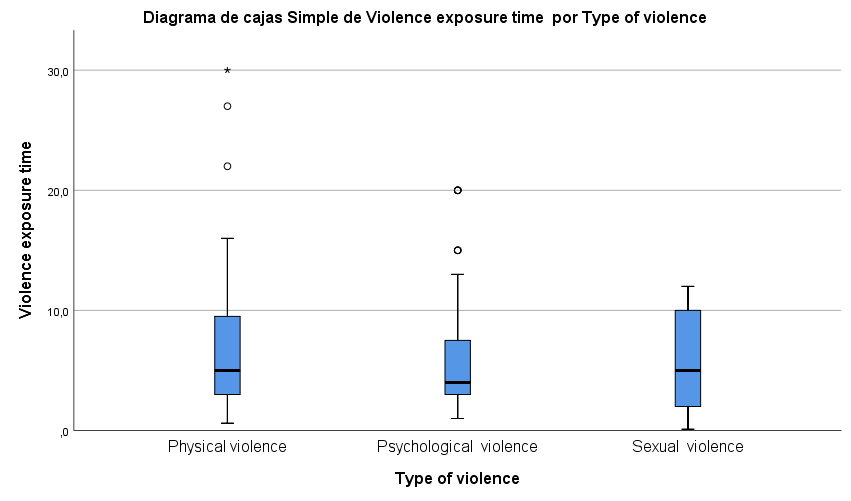


The p-value obtained is greater than 0.05 (p value>0.05), so there are no significant differences between the types of violence, in relation to the times of exposure to violence (Table 5).

**Table 5**

*Post hoc Test violence exposure time- type of violence*

| **Multiple comparisons** | | | | | | |
| --- | --- | --- | --- | --- | --- | --- |
| Dependent variable: | Violence exposure time | | | | | |
| HSD Tukey | | | | | | |
| (I) Type of violence | | Difference of means (I-J) | Error deviation | Sig. | Confidence interval 95% | |
|  |  |  |  |  | Lower limit | Upper limit |
| Physical violence | Psychological violence | 0,9407 | 1,1026 | 0,671 | -1,677 | 3,558 |
|  | Sexual violence | 1,4881 | 1,8212 | 0,693 | -2,835 | 5,811 |
| Psychological violence | Physical violence | -0,9407 | 1,1026 | 0,671 | -3,558 | 1,677 |
|  | Sexual violence | 0,5474 | 1,8335 | 0,952 | -3,805 | 4,900 |
| Sexual violence | Physical violence | -1,4881 | 1,8212 | 0,693 | -5,811 | 2,835 |
|  | Psychological violence | -0,5474 | 1,8335 | 0,952 | -4,900 | 3,805 |

**Figure 4**

*Marital status (N; %)*

The p-value obtained is greater than 0.05 (p value>0.05), so there are no significant differences between the percentages of the types of violence by marital status. There is the following post hoc test (Chi Square) (Table 6).

**Table 6**

*Post hoc differences between the percentages of the types of violence by marital status*

| **Value in table** | **Adjusted_z_score** | **Chi_square** | **p_value** | **Observation** |
| --- | --- | --- | --- | --- |
| 6 | -0,7895 | 0,62331 | 0,430 | It is not significat |
| 7 | 0,0361 | 0,0013 | 0,971 | It is not significat |
| 3 | 1,2532 | 1,57051 | 0,210 | It is not significat |
| 19 | 0,4983 | 0,2483 | 0,618 | It is not significat |
| 15 | -0,5808 | 0,33733 | 0,561 | It is not significat |
| 4 | 0,1308 | 0,01711 | 0,896 | It is not significat |
| 5 | 1,3533 | 1,83142 | 0,176 | It is not significat |
| 1 | -1,5982 | 2,55424 | 0,110 | It is not significat |
| 1 | 0,3895 | 0,15171 | 0,697 | It is not significat |
| 26 | -0,5612 | 0,31495 | 0,575 | It is not significat |
| 29 | 1,2651 | 1,60048 | 0,206 | It is not significat |
| 4 | -1,1565 | 1,33749 | 0,248 | It is not significat |

**Figure 5**

*Age Group (N, %)*

The p-value obtained is greater than 0.05 (p value>0.05), so there are no significant differences between the percentages of the types of violence by Age Group. There is the following post hoc test (Chi Square) (Table 7).

**Table 7**

*Post hoc test differences between the percentages of the types of violence by Age Group.*

| **Valor en tabla** | **adjusted_z_score** | **Chi_square** | **p_value** | **Observación** |
| --- | --- | --- | --- | --- |
| 15 | -1,1 | 1,2100 | 0,271 | It is not significat |
| 18 | 0,6 | 0,3600 | 0,549 | It is not significat |
| 5 | 0,8 | 0,6400 | 0,424 | It is not significat |
| 41 | 1,1 | 1,2100 | 0,271 | It is not significat |
| 34 | -0,6 | 0,3600 | 0,549 | It is not significat |
| 7 | -0,8 | 0,6400 | 0,424 | It is not significat |

**Appendix 3**

**Table 8**

*Differences between groups of cognitive, clinical and sociodemographic variables*

*Post hoc Test*

| **Multiple comparisons** | | | | | | | |
| --- | --- | --- | --- | --- | --- | --- | --- |
| HSD Tukey | | | | | | | |
| Independen variable | | | Difference of means (I-J) | Error deviation | Sig. | Confidence interval 95% | |
|  |  |  |  |  |  | Lower limit | Upper limit |
| NART | Physical violence | Psychological violence | -0,9 | 1,0 | 0,636 | -3,4 | 1,5 |
|  |  | Sexual violence | -5,3 | 1,7 | 0,007 | -9,4 | -1,2 |
|  | Psychological violence | Physical violence | 0,9 | 1,0 | 0,636 | -1,5 | 3,4 |
|  |  | Sexual violence | -4,3 | 1,7 | 0,036 | -8,4 | -0,2 |
|  | Sexual violence | Physical violence | 5,3 | 1,7 | 0,007 | 1,2 | 9,4 |
|  |  | Psychological violence | 4,3 | 1,7 | 0,036 | 0,2 | 8,4 |
| RAVLT | Physical violence | Psychological violence | -0,1 | 0,2 | 0,733 | -0,6 | 0,3 |
|  |  | Sexual violence | -0,1 | 0,3 | 0,964 | -0,8 | 0,6 |
|  | Psychological violence | Physical violence | 0,1 | 0,2 | 0,733 | -0,3 | 0,6 |
|  |  | Sexual violence | 0,1 | 0,3 | 0,979 | -0,7 | 0,8 |
|  | Sexual violence | Physical violence | 0,1 | 0,3 | 0,964 | -0,6 | 0,8 |
|  |  | Psychological violence | -0,1 | 0,3 | 0,979 | -0,8 | 0,7 |
| Indirect digits | Physical violence | Psychological violence | -0,1 | 0,1 | 0,748 | -0,4 | 0,2 |
|  |  | Sexual violence | -0,2 | 0,2 | 0,547 | -0,7 | 0,3 |
|  | Psychological violence | Physical violence | 0,1 | 0,1 | 0,748 | -0,2 | 0,4 |
|  |  | Sexual violence | -0,1 | 0,2 | 0,817 | -0,7 | 0,4 |
|  | Sexual violence | Physical violence | 0,2 | 0,2 | 0,547 | -0,3 | 0,7 |
|  |  | Psychological violence | 0,1 | 0,2 | 0,817 | -0,4 | 0,7 |
| Direct digits | Physical violence | Psychological violence | 0,0 | 0,1 | 0,922 | -0,3 | 0,2 |
|  |  | Sexual violence | -0,1 | 0,2 | 0,936 | -0,5 | 0,4 |
|  | Psychological violence | Physical violence | 0,0 | 0,1 | 0,922 | -0,2 | 0,3 |
|  |  | Sexual violence | 0,0 | 0,2 | 0,993 | -0,5 | 0,4 |
|  | Sexual violence | Physical violence | 0,1 | 0,2 | 0,936 | -0,4 | 0,5 |
|  |  | Psychological violence | 0,0 | 0,2 | 0,993 | -0,4 | 0,5 |
| D2 | Physical violence | Psychological violence | 0,3 | 0,2 | 0,383 | -0,2 | 0,8 |
|  |  | Sexual violence | 0,8 | 0,4 | 0,088 | -0,1 | 1,7 |
|  | Psychological violence | Physical violence | -0,3 | 0,2 | 0,383 | -0,8 | 0,2 |
|  |  | Sexual violence | 0,5 | 0,4 | 0,388 | -0,4 | 1,4 |
|  | Sexual violence | Physical violence | -0,8 | 0,4 | 0,088 | -1,7 | 0,1 |
|  |  | Psychological violence | -0,5 | 0,4 | 0,388 | -1,4 | 0,4 |
| Coding | Physical violence | Psychological violence | -0,1 | 0,2 | 0,835 | -0,6 | 0,3 |
|  |  | Sexual violence | 0,0 | 0,3 | 1,000 | -0,7 | 0,7 |
|  | Psychological violence | Physical violence | 0,1 | 0,2 | 0,835 | -0,3 | 0,6 |
|  |  | Sexual violence | 0,1 | 0,3 | 0,937 | -0,6 | 0,9 |
|  | Sexual violence | Physical violence | 0,0 | 0,3 | 1,000 | -0,7 | 0,7 |
|  |  | Psychological violence | -0,1 | 0,3 | 0,937 | -0,9 | 0,6 |
| TMT-A | Physical violence | Psychological violence | -0,8 | 0,6 | 0,359 | -2,1 | 0,6 |
|  |  | Sexual violence | 0,1 | 0,9 | 0,992 | -2,1 | 2,3 |
|  | Psychological violence | Physical violence | 0,8 | 0,6 | 0,359 | -0,6 | 2,1 |
|  |  | Sexual violence | 0,9 | 0,9 | 0,612 | -1,3 | 3,1 |
|  | Sexual violence | Physical violence | -0,1 | 0,9 | 0,992 | -2,3 | 2,1 |
|  |  | Psychological violence | -0,9 | 0,9 | 0,612 | -3,1 | 1,3 |
| TMT-B | Physical violence | Psychological violence | -0,5 | 0,3 | 0,370 | -1,3 | 0,4 |
|  |  | Sexual violence | 0,2 | 0,6 | 0,955 | -1,2 | 1,5 |
|  | Psychological violence | Physical violence | 0,5 | 0,3 | 0,370 | -0,4 | 1,3 |
|  |  | Sexual violence | 0,6 | 0,6 | 0,517 | -0,7 | 2,0 |
|  | Sexual violence | Physical violence | -0,2 | 0,6 | 0,955 | -1,5 | 1,2 |
|  |  | Psychological violence | -0,6 | 0,6 | 0,517 | -2,0 | 0,7 |
| STROOP | Physical violence | Psychological violence | 0,0 | 0,2 | 0,960 | -0,4 | 0,3 |
|  |  | Sexual violence | -0,2 | 0,3 | 0,709 | -0,9 | 0,4 |
|  | Psychological violence | Physical violence | 0,0 | 0,2 | 0,960 | -0,3 | 0,4 |
|  |  | Sexual violence | -0,2 | 0,3 | 0,808 | -0,8 | 0,5 |
|  | Sexual violence | Physical violence | 0,2 | 0,3 | 0,709 | -0,4 | 0,9 |
|  |  | Psychological violence | 0,2 | 0,3 | 0,808 | -0,5 | 0,8 |
| FVFT/TFVF | Physical violence | Psychological violence | -0,1 | 0,1 | 0,767 | -0,5 | 0,2 |
|  |  | Sexual violence | -0,4 | 0,2 | 0,293 | -0,9 | 0,2 |
|  | Psychological violence | Physical violence | 0,1 | 0,1 | 0,767 | -0,2 | 0,5 |
|  |  | Sexual violence | -0,3 | 0,2 | 0,531 | -0,9 | 0,3 |
|  | Sexual violence | Physical violence | 0,4 | 0,2 | 0,293 | -0,2 | 0,9 |
|  |  | Psychological violence | 0,3 | 0,2 | 0,531 | -0,3 | 0,9 |
| SVFT/TFVS | Physical violence | Psychological violence | -0,3 | 0,2 | 0,472 | -0,8 | 0,3 |
|  |  | Sexual violence | -0,4 | 0,4 | 0,629 | -1,3 | 0,6 |
|  | Psychological violence | Physical violence | 0,3 | 0,2 | 0,472 | -0,3 | 0,8 |
|  |  | Sexual violence | -0,1 | 0,4 | 0,976 | -1,0 | 0,8 |
|  | Sexual violence | Physical violence | 0,4 | 0,4 | 0,629 | -0,6 | 1,3 |
|  |  | Psychological violence | 0,1 | 0,4 | 0,976 | -0,8 | 1,0 |
| HADS- A | Physical violence | Psychological violence | -1,0 | 0,8 | 0,373 | -2,9 | 0,8 |
|  |  | Sexual violence | -1,6 | 1,3 | 0,410 | -4,7 | 1,4 |
|  | Psychological violence | Physical violence | 1,0 | 0,8 | 0,373 | -0,8 | 2,9 |
|  |  | Sexual violence | -0,6 | 1,3 | 0,890 | -3,7 | 2,5 |
|  | Sexual violence | Physical violence | 1,6 | 1,3 | 0,410 | -1,4 | 4,7 |
|  |  | Psychological violence | 0,6 | 1,3 | 0,890 | -2,5 | 3,7 |
| HADS -D | Physical violence | Psychological violence | -0,3 | 0,8 | 0,932 | -2,2 | 1,6 |
|  |  | Sexual violence | -1,7 | 1,3 | 0,394 | -4,8 | 1,4 |
|  | Psychological violence | Physical violence | 0,3 | 0,8 | 0,932 | -1,6 | 2,2 |
|  |  | Sexual violence | -1,4 | 1,3 | 0,526 | -4,6 | 1,7 |
|  | Sexual violence | Physical violence | 1,7 | 1,3 | 0,394 | -1,4 | 4,8 |
|  |  | Psychological violence | 1,4 | 1,3 | 0,526 | -1,7 | 4,6 |
| MILLON | Physical violence | Psychological violence | -7,5 | 4,4 | 0,205 | -17,9 | 2,9 |
|  |  | Sexual violence | -13,2 | 7,2 | 0,163 | -30,4 | 3,9 |
|  | Psychological violence | Physical violence | 7,5 | 4,4 | 0,205 | -2,9 | 17,9 |
|  |  | Sexual violence | -5,8 | 7,3 | 0,709 | -23,0 | 11,5 |
|  | Sexual violence | Physical violence | 13,2 | 7,2 | 0,163 | -3,9 | 30,4 |
|  |  | Psychological violence | 5,8 | 7,3 | 0,709 | -11,5 | 23,0 |
| PCL-5 | Physical violence | Psychological violence | 0,3 | 1,5 | 0,979 | -3,4 | 4,0 |
|  |  | Sexual violence | 0,6 | 2,6 | 0,970 | -5,5 | 6,7 |
|  | Psychological violence | Physical violence | -0,3 | 1,5 | 0,979 | -4,0 | 3,4 |
|  |  | Sexual violence | 0,3 | 2,6 | 0,992 | -5,8 | 6,4 |
|  | Sexual violence | Physical violence | -0,6 | 2,6 | 0,970 | -6,7 | 5,5 |
|  |  | Psychological violence | -0,3 | 2,6 | 0,992 | -6,4 | 5,8 |
| GENCAT | Physical violence | Psychological violence | 0,6 | 3,1 | 0,979 | -6,7 | 7,9 |
|  |  | Sexual violence | 1,2 | 5,1 | 0,970 | -10,9 | 13,3 |
|  | Psychological violence | Physical violence | -0,6 | 3,1 | 0,979 | -7,9 | 6,7 |
|  |  | Sexual violence | 0,6 | 5,1 | 0,992 | -11,6 | 12,8 |
|  | Sexual violence | Physical violence | -1,2 | 5,1 | 0,970 | -13,3 | 10,9 |
|  |  | Psychological violence | -0,6 | 5,1 | 0,992 | -12,8 | 11,6 |
